# Interpreting COVID-19 deaths among nursing home residents in the US: The changing role of facility quality over time

**DOI:** 10.1371/journal.pone.0256767

**Published:** 2021-09-01

**Authors:** Debasree Das Gupta, Uma Kelekar, Sidney C. Turner, Anupam A. Sule, Taya G. Jerman

**Affiliations:** 1 Department of Kinesiology and Health Science, Emma Eccles Jones College of Education and Human Services, Utah State University, Logan, Utah, United States of America; 2 School of Business, College of Business, Innovation, Leadership and Technology, Marymount University, Arlington, Virginia, United States of America; 3 Fors Marsh Group, Arlington, Virginia, United States of America; 4 St. Joseph Mercy Oakland, Pontiac, Michigan, United States of America; China University of Mining and Technology, CHINA

## Abstract

A report published last year by the Centers for Medicare & Medicaid Services (CMS) highlighted that COVID-19 case counts are more likely to be high in lower quality nursing homes than in higher quality ones. Since then, multiple studies have examined this association with a handful also exploring the role of facility quality in explaining resident deaths from the virus. Despite this wide interest, no previous study has investigated how the relation between quality and COVID-19 mortality among nursing home residents may have changed, if at all, over the progression of the pandemic. This understanding is indeed lacking given that prior studies are either cross-sectional or are analyses limited to one specific state or region of the country. To address this gap, we analyzed changes in nursing home resident deaths across the US between June 1, 2020 and January 31, 2021 (n = 12,415 nursing homes X 8 months) using both descriptive and multivariable statistics. We merged publicly available data from multiple federal agencies with mortality rate (per 100,000 residents) as the outcome and CMS 5-star quality rating as the primary explanatory variable of interest. Covariates, based on the prior literature, consisted of both facility- and community-level characteristics. Findings from our secondary analysis provide robust evidence of the association between nursing home quality and resident deaths due to the virus diminishing over time. In connection, we discuss plausible reasons, especially duration of staff shortages, that over time might have played a critical role in driving the quality-mortality convergence across nursing homes in the US.

## Introduction

About a year into the COVID-19 pandemic, cases and deaths among nursing homes residents in the US show a downward trend [1] but continue to remain high [2]. In February 2021, one out of every hundred nursing home residents died from this infection, a rate that was half the COVID-19 mortality rate (about 2 per 100 residents) in the previous month of January but double the rate (about 0.5 per 100 residents) in September 2020 [2]. Previous findings, among other things, have highlighted lower quality of care in nursing homes to be a thorny issue impacting risk of death among facility residents [3, 4]. In synergy, multiple recent studies and reports, including one from the Centers for Medicare & Medicaid Services (CMS) [5], reported CMS five-star quality rating to be a significant determinant of cases and deaths among residents [6, 7]. But, as the pandemic progressed, the question that remained unaddressed is whether nursing home quality mattered more or less over time. Evidence in this regard is lacking with no prior studies examining how the role played by this factor may (or may not) have changed across the US over time. Such an interpretation, as indicated by our findings, may be essential for nursing home administrators to understand the challenging role of managing resources, specifically workforce over time during prolonged public health emergencies.

To address the above mentioned gap, we were interested in examining the evolution, if any, in the association between nursing home quality and resident deaths. While prior analyses are mostly focused on interpreting COVID-19 cases and outbreaks across nursing homes, a smaller set reported findings explaining variations in deaths with a few [7–9] linking resident deaths to nursing home quality. He et al. [7] examined data for April-June 2020 on skilled nursing facilities in California and found that nursing homes with 5-star ratings were less likely to have COVID-19 resident deaths after adjusting for facility (size and ownership) and resident (racial: percent white) characteristics. In contrast, three studies reported findings of no relation between quality and COVID-19 deaths in nursing homes. Additionally, for the March-November 2020 period, evidence of a decline in mortality risk of nursing home residents adjusted for patient characteristics is forwarded in Kosar et al. [10] but factors, either facility- or community-level, behind this decrease are not explored. The generalizability of findings in these studies is however limited with data analyzed for a single state [7–9, 11], or from a single multifacility provider with facilities mostly in the Northeast [10], or using descriptive statistics only [9].

In contrast, we conduct statistical analysis using both descriptive and multivariable methods to examine resident deaths from the virus across nursing homes in the US between June 1–2020 and January 31–2021 (n = 12,415 nursing homes X 8 months). We hypothesized that, other things remaining equal, the strength of the relation (association) between nursing home quality and resident COVID-19 deaths will diminish over time. Such a scenario is plausible if, after the initial shock, nursing homes across the board were able to adjust to the pandemic with knowledge (for example, asymptomatic transmission of virus) and best practices accumulating over time. Alternatively, with expanding contagion, challenges of higher quality nursing homes may have started to resemble that experienced by facilities with lower star ratings. If so, any gap in COVID-19 mortality outcomes that may have differentiated higher from lower quality nursing homes may have decreased over time.

## Materials and methods

### Data

We used the publicly available Nursing Home COVID-19 [5] data released on May-26, 2020 and updated weekly by the CMS. After download on March-1, 2021, we retained data for the period between June-1, 2020 and January-31, 2021 and for nursing homes: i) in all states except Guam and Puerto Rico, ii) that passed the CMS quality assurance check, iii) had no missing occupancy information, and iv) reported data for all weeks in our study period. Following this initial data clean, we created facility-level monthly data (n = 12,473 facilities X 8 months) by aggregating the weekly CMS data. We combined this dataset with Nursing Home Compare [5] data and LTCFocus [12] data that respectively include information on facility (such as, 5-star quality rating) and resident characteristics (such as %White residents, %Medicaid). We then merged this facility-month level master dataset with community-level data on urbanity [13], social vulnerability [14], COVID-19 deaths, population [15], and core-based statistical area (CBSA) codes [16]. The final sample size (n = 99,320), after deletion of nonmatched observations from merging of community-level data, consisted of 12,415 facilities over an 8-month period. The unit of observation of this dataset was facility-month. We selected June-1, 2020 as the start of our study period partly to capture information on the 8 full months since data release by the CMS [5] on May 26, 2020. But more importantly, this start date matched with the period for which data reliable for longitudinal analysis was available from the CMS [5].

### Outcome and primary explanatory variable

The progression of the pandemic across communities varied over time. Hence, for the purpose of comparability, the outcome variable in our descriptive analysis is a rate ratio [17] representing nursing home resident mortality rate relative to CBSA mortality rate [18, 19]. The formula for computing this variable is provided in S1 Appendix. In contrast, in our multivariable regression analysis, the outcome variable is nursing home resident COVID-19 death rate (per 100,000 residents) given that this method allows the inclusion of control variables including community conditions related to the pandemic.

The explanatory variable of interest is CMS’s overall 5-star quality rating of nursing homes [20, 21]. Beginning in December 2008, each nursing home participating in Medicare or Medicaid started receiving an overall quality rating with values ranging between 1 (= lowest performing) and 5 (= highest performing) stars from the CMS. CMS creates the 5-star Quality Rating System to guide consumers, their families, and caregivers when comparing nursing homes based on their performance outcomes. The CMS overall star rating that we used in our study is constructed based on extensive areas of assessment ranging from nursing home environment to patient care. Specifically, CMS computes the overall star rating from nursing home performance outcomes in the three areas of: i) health inspections, ii) staffing (staffing ratios in conjunction with patient needs), and iii) other quality measures (comprising of 15 different physical and clinical measures) [20, 21]. Each of these three domains are weighted differently in the computation of the overall star rating, with health inspections rating weighing heavily and based on findings from the most recent 3-year state health surveys [20, 21]. To avoid the possibility that measured nursing home quality is endogenous to COVID-19 outbreak, we used the last available (December 2019) pre-pandemic 5-star rating that enabled us to evaluate how pre-COVID nursing home quality predicted resident COVID-19 mortality outcomes over time.

### Covariates

Prior studies reported significant variations in resident COVID-19 deaths across facility ownership status [7], facility size [7], staffing [11], and resident racial and socioeconomic composition [7, 11, 22, 23]. In our multivariable analysis, we therefore controlled for these facility-level characteristics that included: i) ownership (nominal: government = 0 | not-for-profit = 1 | for-profit = 2), ii) size (ordinal: measured using total number of beds; <50 beds = 0 | 50–99 beds = 1 | 100–199 beds = 2 | 200 beds or more = 3), iii) duration of staff shortage (continuous: measured as the number of weeks in a month that shortages in care personnel comprising of nursing, clinical, aides, and other staffs are reported by a facility), and resident racial and socioeconomic makeup (continuous: measured as % White and % Medicaid residents in a facility). We included three additional facility-level variables which were: personal protective equipment (PPE) supply shortages (continuous: measured as the number of weeks in a month for which shortages in any PPE were reported by a facility), resident confirmed COVID-19 cases, and resident death rate (per 100,000 residents) from all causes excluding COVID-19 as an acuity measure for resident health. Given the evidence that acuity of nursing home residents has been increasing in recent years [24, 25], we opted to use resident death rate from all causes excluding COVID-19 as the proxy indicator for current resident acuity during our study period. We therefore did not use the acuity (case mix) measure variable from the LTCFocus [12] for which data was collected in 2017.

Lastly, we included urbanity (nominal for location: nonmetro = 0 | metro = 1) [13], social vulnerability index (SVI) (continuous: ranging between 0 = lower and 1 = higher vulnerability) [14], and CBSA COVID-19 case and death rates (per 1000,000 population) due to the virus [15] as community-level controls. The SVI is published by the Centers for Disease Control and Prevention [14] and is computed for each county in the US based on 15 social factors, including unemployment, minority status, and disability. We used CBSA codes [16] to identify counties, aggregate corresponding COVID-19 death and population values, and compute death rates for each CBSA. CBSA codes were explicitly defined by the US Office of Management and Budget (OMB) [16] to identify statistical core areas. Each core comprises of a population center closely linked to an adjoining group of counties based on commuting patterns. Consequently, the geographic extent of travel and, thereby infection spread, by workforce and visitors to and from a nursing home are more likely to be encompassed within a CBSA, as opposed to the facility’s county which is a subset of its CBSA. CBSA codes have been used in prior studies examining infectious disease spread [18] as well as to summarize community COVID-19 profiles [19]. For non-metro nursing homes, we used the death rates of the county.

### Statistical analysis

We examined nursing home to CBSA mortality rate ratio using descriptive statistics (median, interquartile range, and trend lines). Our primary analysis involves repeated comparison of nursing homes with different levels of initial quality over multiple months. To account for potential confounders, we apply the multivariable regression methodology. Since the outcome (nursing home resident COVID-19 deaths per 100,000 residents) in this analysis has a distribution similar to a count variable, to account for influential observations that result from the skewed distribution of this dependent variable we first estimated the Poisson specification. To assess the robustness of our baseline results, we also estimated nonzero-inflated (negative binomial) and zero-inflated (zero-inflated Poisson and zero-inflated negative binomial) count model specifications.

The theoretical basis of the zero inflated models is that the data comprises of excess zero outcomes representing, in this case, facilities unexposed and, in turn, not at risk of a death from the disease [26]. Alternatively, zero outcomes of no deaths may also result in the presence of the virus representing true zeros. The zero inflated regression specifications are therefore a two-part model. The logistic part estimates the contribution of factors in explaining whether or not a facility was at risk of death. But, given the risk of a death, the second part (count part) predicts mortality counts for facilities that are not excess zeros. Additional detailed description of each of the count model specifications, including zero-inflated models, are provided in Karaszia et al. [27] and Hardin and Hilbe [28].

For each of the multivariable regression models, we clustered observations by CBSA since the cluster option yields robust standard errors adjusted for nonindependence of mortality outcomes over time and within each CBSA. We performed all statistical analysis using Stata (version 15) and report results from the count model providing the best fit to the data. We interpret statistically significant findings at p ≤ 0.05 (unless specified otherwise).

## Results

### Descriptive analysis

**Fig 1:**
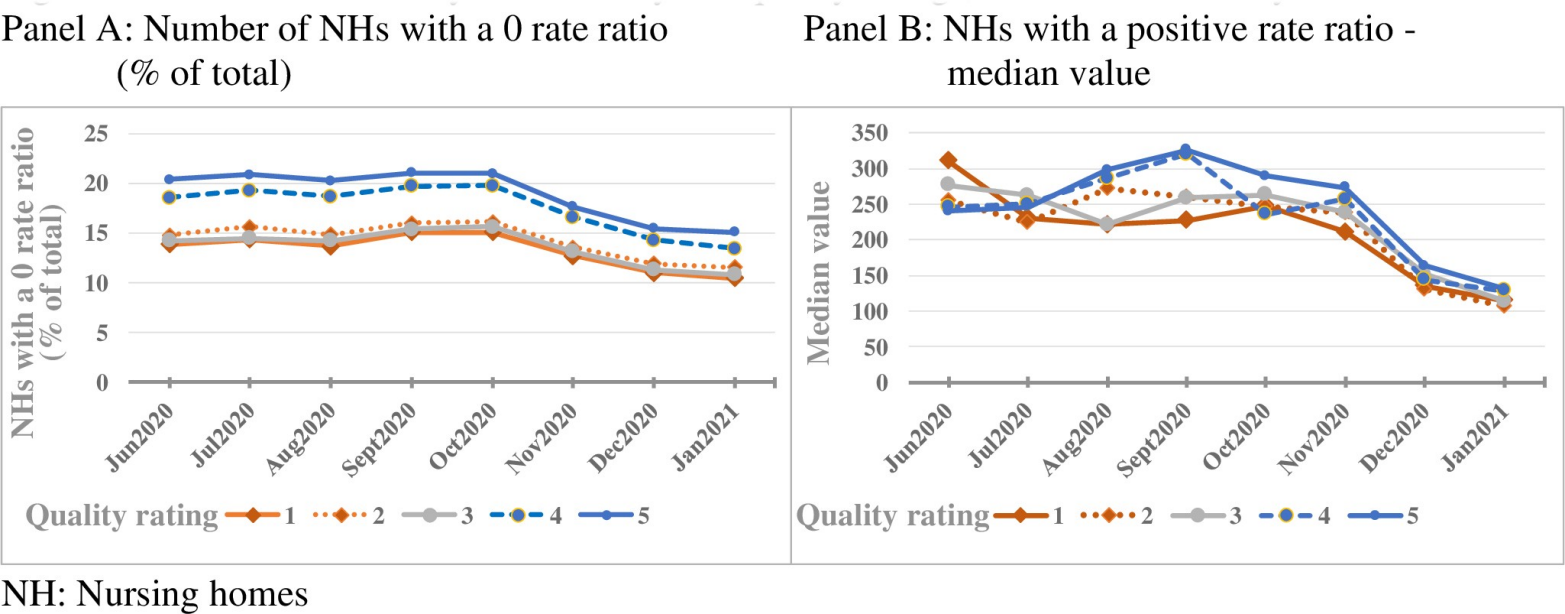
Differences in mortality outcomes by nursing home quality ratings, June 2020—January 2021.

In Table 1, we provide the descriptive statistics on nursing home to CBSA COVID-19 mortality rate ratio in our sample of nursing homes. As is evident from Table 1, all eight months are dominated by nursing homes with a zero rate ratio. For nursing homes with a positive rate ratio, the median over the study period was 190 with all months indicating a highly skewed distribution in the positive direction. The IQR fluctuates but decreases over the later months indicating a less dispersed distribution. A similar pattern of convergence is evident in Fig 1 revealing that the differences in mortality outcomes by nursing home quality ratings reduced over time. Fig 1 also indicates that the nursing home quality-mortality relation may have been different between the zero versus the nonzero mortality outcome nursing homes. Findings from our multivariable analysis further confirmed this difference in mortality trend which we discuss next.

**Table 1:** Descriptive statistics on nursing home to CBSA COVID-19 mortality rate ratio, June 2020—January 2021.

Month	nursing homes		nursing homes with a positive rate ratio				
	Total number	% with 0 rate ratio	Number	% of total	Mean	Median	Interquartile Range (IQR)
Jun 2020-Jan 2021	91,234	77.44	20,578	22.56	397.59	190	336
Jun 2020	10,105	81.91	1,828	18.09	596.46	261	492
Jul 2020	10,803	84.72	1,651	15.28	506.23	238	392
Aug 2020	11,074	81.84	2,015	18.16	497.82	261	420
Sept 2020	11,248	87.30	1,429	12.70	511.07	277	427
Oct 2020	11,595	87.71	1,425	12.29	530.22	254	424
Nov 2020	12,014	73.75	3,154	26.25	464.55	243	411
Dec 2020	12,198	64.08	4,382	35.92	279.44	144	241
Jan 2021	12,197	61.52	4,694	38.48	229.39	120	193

### Multivariable regression analysis

We pooled the monthly data and examined changes in relations over time by interacting the main independent variable, quality rating, as well as covariates with time (month). In Tables 2 and 3, we report the association between nursing home mortality rate (per 100,000 residents) and overall quality rating. While the likelihood ratio test [29] confirmed negative binomial as more appropriate than the Poisson specification, the Vuong test [30, 31] identified the zero inflated negative binomial model to perform better than the standard negative binomial one. Furthermore, the Akaike’s information criteria (AIC) and Bayesian information criteria (BIC) [28] values were the smallest for the zero-inflated negative binomial specification indicating this model to be the best fit compared to the other count models we tested. We therefore report and interpret findings from the zero inflated negative binomial model only.

**Table 2:** Multivariable regression results–role of nursing home quality, parsimonious models.

*Dependent Variable*: nursing home mortality rate (per 100,000 residents)	Parsimonious Models (Adjusted for CBSA case and death rates)^1^					
	Poisson	Zero inflated Poisson		Negative Binomial	Zero Inflated Negative Binomial	
		Count estimation	Logit estimation		Count Estimation	Logit estimation
Quality of Nursing Homes (Overall Star Rating)						
Main effect						
Coefficient	-0.088***	0.038**	0.167***	-0.129***	0.033*	0.167***
SE	0.018	0.017	0.019	0.023	0.018	0.019
CI	-0.123, -0.053	0.006, 0.071	0.129, 0.205	-0.174, -0.084	-0.001, 0.068	0.129, 0.205
Interaction effect						
Coefficient	0.020***	0.001	-0.024***	0.028***	0.002	-0.024***
SE	0.004	0.003		0.005	0.003	0.003
CI	0.013, 0.027	-0.004, 0.007	-0.031, -0.018	0.019, 0.037	-0.004, 0.008	-0.031, -0.018
Model Comparison						
Likelihood ratio Test (χ^2^)^7^				100,000,000***		
Vuong Test (z)^8^					58.86***	
Model fit						
AIC	372,000,000	105,000,000		525,190.6	477,350.7	
BIC	372,000,000	105,000,000		525,275.3	477,510.7	

Note: ^1^ Parsimonious models to examine unadjusted role of nursing home quality (after accounting for the level/progression of the pandemic in the community).

^2^ The full models included all covariates we presented earlier under the Methods section.

^3^ Abbreviations CI: Confidence Interval (95%) | SE: Standard Error (robust).

^4^ * p < 0.1, ** p < 0.05, *** p < 0.01.

^5^ Data extraction and statistical analysis were conducted by the study authors.

^6^ Missing values: < 2% in each month.

^7^ Poisson versus negative binomial.

^8^ Zero inflated negative binomial versus standard negative binomial.

**Table 3:** Multivariable regression results–role of nursing home quality, full models.

*Dependent Variable*: nursing home mortality rate (per 100,000 residents)	Full Model (Adjusted for all covariates)^2^					
	Poisson	Zero inflated Poisson		Negative Binomial	Zero Inflated Negative Binomial	
		Count estimation	Logit estimation		Count Estimation	Logit estimation
Quality of Nursing Homes (Overall Star Rating)						
Main effect						
Coefficient	-0.040*	-0.001	0.064***	-0.133***	-0.015	0.064***
SE	0.023	0.017	0.021	0.029	0.018	0.021
CI	-0.085, 0.005	-0.035, 0.033	0.023, 0.105	-0.191, -0.076	-0.050, 0.020	0.023, 0.105
Interaction effect						
Coefficient	0.011***	0.0006	-0.013***	0.016***	0.0008	-0.013***
SE	0.004	0.003	0.004	0.006	0.003	0.004
CI	0.003, 0.020	-0.006, 0.007	-0.020, -0.006	0.005, 0.027	-0.005, 0.007	-0.020, -0.006
Model Comparison						
Likelihood ratio Test (χ^2^)^7^				78,000,000***		
Vuong Test (z)^8^					51.19***	
Model fit						
AIC	275,000,000	78,300,000		486,375.9	436,947.5	
BIC	275,000,000	78,300,000		486,692.8	437,544.0	

Note: ^1^ Parsimonious models to examine unadjusted role of nursing home quality (after accounting for the level/progression of the pandemic in the community).

^2^ The full models included all covariates we presented earlier under the Methods section.

^3^ Abbreviations CI: Confidence Interval (95%) | SE: Standard Error (robust).

^4^ * p < 0.1, ** p < 0.05, *** p < 0.01.

^5^ Data extraction and statistical analysis were conducted by the study authors.

^6^ Missing values: < 2% in each month.

^7^ Poisson versus negative binomial.

^8^ Zero inflated negative binomial versus standard negative binomial.

Findings from the descriptive analysis (Table 1) suggested an excess of zero mortality outcomes in our sample of nursing homes. The zero inflated negative binomial model (full model in Table 3) indicates that the log odds of being an excessive zero would increase by 0.06 (or 6%; 95% CI: 0.023, 0.105) for every unit increase in quality rating holding other variables constant. However, this relation diminished over time as demonstrated by the negative interaction term (-0.013, 95% CI: -0.020, -0.006) which was statistically significant. Furthermore, the quality-mortality rate association in the count part revealed a negative association between the two variables which also decreased over time (positive interaction term). However, both these latter relations fail to attain statistical significance.

Among the statistically significant covariates (shown in S1 Table), a longer duration of staff shortages and larger size of facilities decreased the log odds of being an excess zero outcome. In the count part, while longer duration of staff shortages indicated higher mortality rates, larger size of facilities was associated with lower mortality rates. Additionally, the staff-mortality relation did not change over time, but the association between facility size and mortality rate diminished over time. Finally, a higher proportion of White residents in a nursing home increased the likelihood of being an excess zero outcome, an association that also diminished over time. Among the community-level covariates, higher CBSA mortality rate and metro location decreased the log odds of being an excess zero outcome with both associations diminishing over time. In addition, the higher the mortality rate was in a CBSA, the higher was the mortality rate in a nursing home located in that CBSA and this positive association did not change over time.

## Discussion

As witnessed during this pandemic, nursing home quality is not a nebulous concept and its impact can be critical in determining life and death in the real world. Our findings (Table 3- zero inflated negative binomial: main effect) revealed that higher quality of nursing homes increased the possibility of belonging in the excess zero group not at risk of death. A related finding was that this association diminished over time (Table 3- zero inflated negative binomial: interaction term) showing the reducing role of quality as the pandemic progressed. Together these findings suggest that higher quality nursing homes were better prepared to handle the pandemic in the earlier stages of our study period. This trend is a reflection of the infrastructure and rapidly adaptable processes in place in these nursing homes that helped them not only achieve a higher quality rating but also may have provided an advantage in dealing with the health care emergency due to COVID-19.

However, with the passage of time, a convergence in mortality between low and high quality nursing homes is indicated by our findings. There are two probable explanations to interpret this trend. Nursing homes with a lower star rating may have implemented best practices as new knowledge and guidelines [32, 33] as well as resources [24, 34] to manage the virus became available over time. For instance, to combat the virus, overhaul in care delivery and practices of nursing homes have been reported [24, 35, 36]. This transition toward best practices may have been incentivized by performance-based metrics rewarding reduction in deaths under the Nursing Home Quality Incentive Payment Program beginning in September, 2020 [24, 34]. Consequently, nursing homes with a lower star rating over time may have been able to match their higher quality counterparts in tackling the pandemic. The alternative explanation could be that the structure and processes built by high quality nursing homes was slowly eroded as the pandemic continued to strain human resources [37, 38] and all nursing homes started to resemble one another in their performance. Specifically, increasingly worsening staff shortages and attrition may have been a factor of particular relevance here [24].

The interaction between nursing home quality and staff shortages was not the focus of our multivariable analysis, but we found descriptive evidence in this regard. We found that chronic staff shortages, although lower, rose faster for higher quality nursing homes (S1 Fig). Staff shortages in high-quality nursing homes might have been more acute on account of the employment of part-time workers by for-profit facilities. In our sample, a majority (58%) of the for-profit nursing homes were higher quality with a star rating of 3 or above. Recent reports are suggestive of for-profit facilities relying on part time workers [39] and struggling to retain their staff over time [40]. Additionally, an increasingly higher intake of COVID-19 patients by for-profit nursing homes without commensurate increase in staffing especially those trained in specialty services [41] may have put further stress resulting in higher staff turnovers.

Findings from our multivariable analysis are also in support of the central but a negative role of staff shortages, other things remaining equal. Duration of staff shortages were significantly associated with mortality rates both in the logit and count parts of the zero inflated negative binomial model. Our results also indicated that the adverse role of staff shortages did not change over time but continued to be an important factor. Throughout our study period, staff shortages (S1 Fig) self-reported by nursing homes persisted at higher levels which has received wide reporting [42] and analysis [43, 44]. An additional factor driving this trend may have been rising demand for nursing services in hospitals. As the pandemic worsened, hospitals turned to expensive staffing agencies to meet their demand [43, 45, 46] thereby generating lucrative travel nursing job opportunities for nursing home nurses. This possibly may have shifted staff from nursing homes to hospitals and further accentuated shortages and worsened the burn-out of staff who remained.

There is longstanding evidence [42, 47–49] of nursing home staff being poorly paid, employed without benefits (such as sick leave or health insurance), and many working multiple jobs [50]. Early evidence indicates innovative approaches adopted in other developed countries, such as requiring nursing home staff to choose a single work location with compensation for the resulting income loss [51] or, offering hazard pay and surge staffing/recruitment were associated with [51, 52] lower cases and deaths from the virus. Similar such policies addressing staff pay would become feasible in the US if nursing homes remain financially stable and healthy, especially given increasing costs but declining revenues across nursing homes due to the pandemic [42, 53]. In connection, the role played by Medicaid reimbursement becomes salient as the major source of funding (about 60%) for nursing homes which does not cover about 20–30% of the actual cost of care [53].

Of the other facility-level factors, ownership and shortage of PPE were not associated with mortality in either the early part of our study period or in the later stages as the pandemic progressed. While supply of PPE has been identified in one study [44] as a factor driving staff shortages in nursing homes, the association between PPE shortage and resident deaths may have been complicated by lack of staff training regarding use/reuse of PPE [54]. But, the unequal burden of the disease borne by the non-White population in the early part of the pandemic was reflected by our finding that higher the proportion of White residents in a facility, the higher the likelihood of being an excess zero nursing home. Additionally, our analysis revealed an interestingly complex relation between facility size and mortality rate. Larger the facility size, lower was the death rate. While this finding is compatible with prior results [7, 55, 56], we found that this inverse association between facility size and death rate diminished over time. In addition, our results suggested that larger facility size lowered the possibility of being an excess zero with this association also diminishing over time. The exact mechanisms driving these seemingly contradictory trends warrant further research, possibly regarding how micro-factors [57, 58] as single- versus multiple-occupancy rooms, designated isolation wards, or physical size and configuration of rooms, and their corresponding roles may have varied by facility size.

Finally, community-level external variables (CBSA death rate and metro location) were significant determinants of nursing home mortality outcomes. However, the social vulnerability of the county that a nursing home was located in, did not show significant association with COVID-19 deaths in the nursing home. Together these findings suggest that the progression of the pandemic, as opposed to prior social conditions, in the community was a salient determinant of nursing home mortality outcomes.

### Limitations

In our analysis, we were interested in the initial differences in death rates between high and low quality nursing homes and their change over time. Since fixed effects would be collinear with initial nursing homing quality, we conducted a pooled analysis after interacting all explanatory variables with time (month) to examine change over our study period of 8 months spanning June 2020-January 2021. To account for the non-independence of observations over time (as a result of repeated measures of death rate for a given nursing home) and space, we estimated robust standard errors clustered on CBSA. After adjusting for data quality and incompatibility across multiple sources, we could analyze a subset (n = 12,415 | about 81%) of the nursing homes across the US. We acknowledge that our findings are therefore representative of this sample of nursing homes only. Moreover, we were unable to include data from the early months of the pandemic as weekly CMS COVID-19 data reliable for longitudinal analysis became available only in the last week of May [5].

## Conclusion

Lower quality of care in nursing homes significantly affects the risk of death among facility residents. However, recent evidence on the relation between nursing home quality and resident COVID-19 deaths remains mixed with findings not generalizable as most of these analyses are single state and/or are descriptive investigations. Furthermore, no prior analysis have examined how the role of quality may (or may not) have changed over time. Our findings contribute to this literature by providing robust evidence of the association between nursing home quality and resident deaths due to the virus over time across the US. We hypothesized that, other things remaining equal, the strength of the relation (association) between nursing home quality and resident COVID-19 deaths will diminish over time. Consistent with this hypothesis, we found that quality rating was a significant factor predicting whether a nursing home was at risk of resident COVID-19 death but this relation diminished over time. In connection, we highlighted the critical role of staff shortages and related policy considerations.

## Supporting information

S1 Appendix Formula for mortality rate ratio. (DOCX)

S1 Table Regression results from the two-part zero inflated negative binomial regression (full model). (DOCX)

S1 Fig Nursing homes self-reporting chronic staff shortages (all weeks of the month) by quality ratings, June 2020—January 2021. (TIF)

## References

[1] Chidambaram P, Garfield R, Neuman T, Levitt L. Is the End of the Long-Term Care Crisis Within Sight? New COVID-19 Cases and Deaths in Long-Term Care Facilities Are Dropping [Internet]. KFF. 2021 [cited 2021 Apr 6]. Available from: https://www.kff.org/policy-watch/is-the-end-of-the-long-term-care-crisis-within-sight-new-covid-19-cases-and-deaths-in-long-term-care-facilities-are-dropping/

[2] Public Policy Institute. COVID-19 Nursing Home Resident and Staff Deaths: AARP Nursing Home Dashboard [Internet]. AARP. [cited 2021 Apr 6]. Available from: https://www.aarp.org/ppi/issues/caregiving/info-2020/nursing-home-covid-dashboard.html

[3] Institute of Medicine. Improving the quality of long-term care. Washington, DC: National Academy of Sciences; 2001.

[4] Wiener JM. An Assessment of Strategies for Improving Quality of Care in Nursing Homes. Gerontologist. 2003 Apr 1;43(suppl_2):19–27. doi: 10.1093/geront/43.suppl_2.19 12711721

[5] CMS—Division of Nursing Homes/Quality, Safety, and Oversight Group/Center for Clinical Standards and Quality. COVID-19 Nursing Home Dataset [Internet]. Data.CMS.gov. 2021. Available from: https://data.cms.gov/Special-Programs-Initiatives-COVID-19-Nursing-Home/COVID-19-Nursing-Home-Dataset/s2uc-8wxp

[6] Figueroa JF, Wadhera RK, Papanicolas I, Riley K, Zheng J, Orav EJ, et al. Association of Nursing Home Ratings on Health Inspections, Quality of Care, and Nurse Staffing With COVID-19 Cases. JAMA. 2020 Sep 15;324(11):1103. doi: 10.1001/jama.2020.14709 32790822 PMC7418040

[7] He M, Li Y, Fang F. Is There a Link between Nursing Home Reported Quality and COVID-19 Cases? Evidence from California Skilled Nursing Facilities. J Am Med Dir Assoc. 2020 Jul 1;21(7):905–8. doi: 10.1016/j.jamda.2020.06.016 32674817 PMC7294249

[8] Rowan P, Gupta R, Lester R, Levere M, Liao K, Libersky J, et al. A Study of the COVID-19 Outbreak and Response in Connecticut Long-Term Care Facilities: Final Report [Internet]. Princeton, NJ: Mathematica; 2020 Sep [cited 2021 May 4]. Available from: https://www.mathematica.org/our-publications-and-findings/publications/fr-a-study-of-the-covid-19-outbreak-and-response-in-connecticut-long-term-care-facilities

[9] New York State Department of Health. Factors Associated with Nursing Home Infections and Fatalities in New York State During the COVID-19 Global Health Crisis [Internet]. 2021. Available from: https://www.health.ny.gov/press/releases/2020/docs/nh_factors_report.pdf

[10] Kosar CM, White EM, Feifer RA, Blackman C, Gravenstein S, Panagiotou OA, et al. COVID-19 Mortality Rates Among Nursing Home Residents Declined From March To November 2020. Health Affairs. 2021 Mar 11;40(4):655–63. doi: 10.1377/hlthaff.2020.02191 33705204 PMC8045482

[11] Li Y, Temkin-Greener H, Shan G, Cai X. COVID-19 Infections and Deaths among Connecticut Nursing Home Residents: Facility Correlates. J Am Geriatr Soc. 2020 Sep;68(9):1899–906. doi: 10.1111/jgs.16689 32557542 PMC7323378

[12] Long-term Care. LTCFocus [Internet]. LTC Focus. date unknown [cited 2021 Apr 9]. Available from: https://ltcfocus.org

[13] United States Department of Agriculture. USDA ERS—Rural-Urban Commuting Area Codes [Internet]. USDA Economic Research Service. 2020 [cited 2021 May 6]. Available from: https://www.ers.usda.gov/data-products/rural-urban-commuting-area-codes/

[14] Centers for Disease Control and Prevention. CDC/ATSDR SVI Data and Documentation Download | Place and Health | ATSDR [Internet]. Place and Heath Data & Documentation Download. 2018 [cited 2021 May 4]. Available from: https://www.atsdr.cdc.gov/placeandhealth/svi/data_documentation_download.html

[15] USA Facts. US COVID-19 cases and deaths by state [Internet]. USAFacts.org. 2021 [cited 2021 Apr 6]. Available from: /visualizations/coronavirus-covid-19-spread-map/

[16] Office of Management and Budget. Standards for Delineating Metropolitan and Micropolitan Statistical Areas. Notice Federal Register. 2010 Jun 28;75(123).

[17] Centers for Disease Control and Prevention. Principles of Epidemiology: Home|Self-Study Course SS1978|CDC [Internet]. 2019 [cited 2021 Apr 9]. Available from: https://www.cdc.gov/csels/dsepd/ss1978/index.html

[18] Dahlgren FS, Shay DK, Izurieta HS, Forshee RA, Wernecke M, Chillarige Y, et al. Patterns of seasonal influenza activity in U.S. core-based statistical areas, described using prescriptions of oseltamivir in Medicare claims data. Epidemics. 2019 Mar 1;26:23–31. doi: 10.1016/j.epidem.2018.08.002 30249390 PMC6519085

[19] Centers for Disease Control and Prevention. COVID Data Tracker [Internet]. Centers for Disease Control and Prevention. 2020 [cited 2021 May 4]. Available from: https://covid.cdc.gov/covid-data-tracker

[20] Centers for Medicare and Medicaid Services. Design for Care Compare Nursing Home Five-Star Quality Rating System: [Internet]. 2020 [cited 2021 Apr 6]. Available from: https://www.cms.gov/Medicare/Provider-Enrollment-and-Certification/CertificationandComplianc/Downloads/usersguide.pdf

[21] Centers for Medicare and Medicaid Services. Design for Care Compare Nursing Home Five-Star Quality Rating System: [Internet]. 2021 [cited 2021 Apr 6]. Available from: https://www.cms.gov/Medicare/Provider-Enrollment-and-Certification/CertificationandComplianc/Downloads/usersguide.pdf

[22] Travers JL, Agarwal M, Estrada LV, Dick AW, Gracner T, Wu B, et al. Assessment of Coronavirus Disease 2019 Infection and Mortality Rates Among Nursing Homes With Different Proportions of Black Residents. J Am Med Dir Assoc. 2021 Apr 1;22(4):893-898.e2. 10.1016/j.jamda.2021.02.014 PMC7898962 33762185

[23] Gorges RJ, Konetzka RT. Factors Associated With Racial Differences in Deaths Among Nursing Home Residents With COVID-19 Infection in the US. JAMA Netw Open. 2021 Feb 10;4(2):e2037431–e2037431. doi: 10.1001/jamanetworkopen.2020.37431 33566110 PMC7876590

[24] Denny-Brown N, Stone D, Hays B, Gallaghe D. COVID-19 Intensifies Nursing Home Workforce Challenges [Internet]. Offices of the Assistant Secretary for Planning and Evaluation; 2020 Oct [cited 2021 Jul 19] p. 45. Available from: https://aspe.hhs.gov/reports/covid-19-intensifies-nursing-home-workforce-challenges-0

[25] Grabowski D. Testimony of David C. GRabowski, PhD, Not Forgotten: Protecting Americans from Abuse and Neglect in Nursing Homes [Internet]. Sect. United States Senate Committee on Finance Harvard Medical School, Boston, MA; Mar 6, 2019 p. 15. Available from: https://www.finance.senate.gov/imo/media/doc/Grabowski%20Senate%20Finance%20testimony%20FINAL.pdf

[26] He H, Tang W, Wang W, Crits-Cristoph P. Structural zeroes and zero-inflated models. Shanghai Arch Psychiatry. 2014 Aug;26(4):236–42. doi: 10.3969/j.issn.1002-0829.2014.04.008 25317011 PMC4194007

[27] Karazsia BT, van Dulmen MHM. Regression Models for Count Data: Illustrations using Longitudinal Predictors of Childhood Injury*. J Pediatr Psychol. 2008 Nov 1;33(10):1076–84. doi: 10.1093/jpepsy/jsn055 18522994

[28] Hardin JW, Hilbe JM. Regression Models for Count Data Based on the Negative Binomial(p) Distribution. Stata J. 2014 Jun 1;14(2):280–91.

[29] Yang Z, Hardin JW, Addy CL, Vuong QH. Testing Approaches for Overdispersion in Poisson Regression versus the Generalized Poisson Model. Biom J. 2007 Aug 27;49(4):565–84. doi: 10.1002/bimj.200610340 17638291

[30] Desmarais B, Harden J. Testing for zero inflation in count models: Bias correction for the Vuong test. Stata J. 2013;13(4):810–35.

[31] Vuong QH. Likelihood ratio tests for model selection and non-nested hyptheses. Econometrica. 1989;57:307–33.

[32] Centers for Disease Control and Prevention. Nursing Homes & Long-Term Care [Internet]. Centers for Disease Control and Prevention. 2020 [cited 2021 May 4]. Available from: https://www.cdc.gov/coronavirus/2019-ncov/hcp/nursing-home-long-term-care.html

[33] Rios P, Radhakrishnan A, Williams C, Ramkissoon N, Pham B, Cormack GV, et al. Preventing the transmission of COVID-19 and other coronaviruses in older adults aged 60 years and above living in long-term care: a rapid review. Syst Rev [Internet]. 2020 Sep 25 [cited 2021 May 4];9. Available from: https://www.ncbi.nlm.nih.gov/pmc/articles/PMC7517751/ doi: 10.1186/s13643-020-01486-4 32977848 PMC7517751

[34] US Department of Health and Human Services. Trump Administration Distributes Incentive Payments to Nursing Homes Curbing COVID-19 Deaths and Infections. HHS.gov [Internet]. 2020 Oct 28 [cited 2021 Jul 19]; Available from: https://www.hhs.gov/about/news/2020/10/28/trump-administration-distributes-incentive-payments-to-nursing-homes-curbing-covid-19-deaths-and-infections.html

[35] Miceli S. How Nursing Homes Are Handling COVID-19: Best Practices from Maryland and Massachusetts [Internet]. The National Academies of Sciences, Engineering, and Medicine. 2020. Available from: https://www.nationalacademies.org/news/2020/04/how-nursing-homes-are-handling-covid-19-best-practices-from-maryland-and-massachusetts

[36] Carey S. Winning Practices as COVID-19 Evolves in Nursing Homes [Internet]. The Joint Commission. 2020 [cited 2021 Jul 19]. Available from: https://www.jointcommission.org/resources/news-and-multimedia/blogs/quality-in-nursing-center-care/2020/11/02/Winning Practices as COVID-19 Evolves in Nursing Homes

[37] Bartoldus E, Blumenstock C, Falchuk A. Pandemic of Grief: A Mental Health Challenge for Nursing Home Staff [Internet]. Mental Health America. 2020 [cited 2021 May 4]. Available from: https://www.mhanational.org/blog/pandemic-grief-mental-health-challenge-nursing-home-staff

[38] Mo S, Shi J. The Psychological Consequences of the COVID-19 on Residents and Staff in Nursing Homes. Work Aging Retire. 2020 Oct;6(4):254–9. doi: 10.1093/workar/waaa021 34192005 PMC7665707

[39] Brownlee S, Lenzer J. How Mistreating Nursing Home Staff Helped Spread Covid-19 | Washington Monthly [Internet]. Washington Monthly. 2021 [cited 2021 May 4]. Available from: https://washingtonmonthly.com/2021/04/16/how-mistreating-nursing-home-staff-helped-spread-covid-19/

[40] Gandhi A, Yu H, Grabowski DC. High Nursing Staff Turnover In Nursing Homes Offers Important Quality Information. Health Aff (Millwood). 2021 Mar 1;40(3):384–91. doi: 10.1377/hlthaff.2020.00957 33646872 PMC7992115

[41] Englund W, Jacobs J. How government incentives shaped the nursing home business—and left it vulnerable to a pandemic [Internet]. The Washington Post. 2020 [cited 2021 May 4]. Available from: https://www.washingtonpost.com/business/2020/11/27/nursing-home-incentives/

[42] Soergel A. Where Have Billions in COVID Aid for Nursing Homes Gone? [Internet]. AARP. [cited 2021 May 6]. Available from: https://www.aarp.org/caregiving/health/info-2020/nursing-home-covid-federal-aid-transparency.html

[43] Gibson DM, Greene J. State Actions and Shortages of Personal Protective Equipment and Staff in U.S. Nursing Homes. J Am Geriatr Soc. 2020;68(12):2721–6. doi: 10.1111/jgs.16883 33022757 PMC7675486

[44] Xu H, Intrator O, Bowblis JR. Shortages of Staff in Nursing Homes During the COVID-19 Pandemic: What are the Driving Factors? J Am Med Dir Assoc. 2020 Oct 1;21(10):1371–7. doi: 10.1016/j.jamda.2020.08.002 32981663 PMC7418696

[45] Frey M. Hospitals battle burnout, compete for nurses as pandemic spurs US staffing woes | S&P Global Market Intelligence [Internet]. S&P Global Market Intelligence. 2021 [cited 2021 May 4]. Available from: https://www.spglobal.com/marketintelligence/en/news-insights/latest-news-headlines/hospitals-battle-burnout-compete-for-nurses-as-pandemic-spurs-us-staffing-woes-63316216

[46] Wells K. New York is offering nurses up to $7K a week. Michigan is offering way less. [Internet]. Michigan Radio. 2020 [cited 2021 May 4]. Available from: https://www.michiganradio.org/post/new-york-offering-nurses-7k-week-michigan-offering-way-less

[47] Eaton J. Nursing Home Workers Face Danger During Coronavirus [Internet]. AARP. 2020 [cited 2021 May 6]. Available from: https://www.aarp.org/caregiving/health/info-2020/nursing-home-workers-during-coronavirus.html

[48] Cohen-Mansfield J. Turnover among nursing home staff. A review. Nurs Manage. 1997 May;28(5):59–62, 64. 9287799

[49] Barnett ML, Grabowski DC. Nursing Homes Are Ground Zero for COVID-19 Pandemic. JAMA Health Forum. 2020 Mar 24;1(3):e200369. 10.1001/jamahealthforum.2020.0369 36218601

[50] Baughman RA, Stanley B, Smith KE. Second Job Holding Among Direct Care Workers and Nurses: Implications for COVID-19 Transmission in Long-Term Care. Med Care Res Rev. 2020 Nov 19;1077558720974129. doi: 10.1177/1077558720974129 33213282

[51] Silverman M, Clarke M, Stranges S. Did Lessons From SARS Help Canada’s Response to COVID-19? Am J Public Health. 2020 Nov 12;110(12):1797–9. doi: 10.2105/AJPH.2020.305936 33180584 PMC7661989

[52] Canadian Institute for Health Information. Pandemic Experience in the Long-Term Care Sector: How Does Canada Compare With Other Countries? CIHI; 2020.

[53] American Health Care Association, National Center for Assisted Living. Survey: Nursing Homes Incurring Significant Costs and Financial Hardship in Response to COVID-19 [Internet]. 2020 [cited 2021 Apr 25]. Available from: https://www.ahcancal.org/News-and-Communications/Fact-Sheets/FactSheets/Survey-SNF-COVID-Costs.pdf

[54] MITRE Corporation. Coronavirus Commission for Safety and Quality in Nursing Homes [Internet]. 2020 Sep [cited 2021 Apr 25]. Available from: https://sites.mitre.org/nhcovidcomm/wp-content/uploads/sites/14/2020/09/FINAL-REPORT-of-NH-Commission-Public-Release-Case-20-2378.pdf

[55] Unruh MA, Yun H, Zhang Y, Braun RT, Jung H-Y. Nursing Home Characteristics Associated With COVID-19 Deaths in Connecticut, New Jersey, and New York. J Am Med Dir Assoc. 2020 Jul 1;21(7):1001–3. doi: 10.1016/j.jamda.2020.06.019 32674812 PMC7294277

[56] Spurlock B, Harrington C. COVID-19 in California’s Nursing Homes: Factors Associated with Cases and Deaths [Internet]. California Health Care Foundation. 2020 [cited 2021 May 4]. Available from: https://www.chcf.org/publication/covid-19-californias-nursing-homes-factors-cases-deaths/

[57] Cohen LW, Zimmerman S, Reed D, Brown P, Bowers BJ, Nolet K, et al. The Green House Model of Nursing Home Care in Design and Implementation. Health Serv Res. 2016 Feb;51(Suppl 1):352–77. doi: 10.1111/1475-6773.12418 26601799 PMC5338211

[58] Anderson DC, Grey T, Kennelly S, O’Neill D. Nursing Home Design and COVID-19: Balancing Infection Control, Quality of Life, and Resilience. J Am Med Dir Assoc. 2020 Nov 1;21(11):1519–24. doi: 10.1016/j.jamda.2020.09.005 33138934 PMC7603995

