# Supplementary material for: Interpreting COVID-19 deaths among nursing home residents in the US: The changing role of facility quality over time
Source: PLoS One. 2021 Sep 1;16(9):e0256767. doi: 10.1371/journal.pone.0256767 (PMC8409689; doi:10.1371/journal.pone.0256767)
Supplement: S1 Appendix — (DOCX) [file pone.0256767.s001.docx]

**S1 Appendix: - Formula for mortality rate ratio**

The formula for calculating COVID-19 mortality rate ratio for the descriptive analysis:

$$\left[ \text{Y}_{\text{ij}} \right]_{\text{CBSA}_{\text{k}}}$$

$\text{=}\frac{\text{COVID19 death rate}_{\text{Nursing home residents}}}{\text{COVID19 death rate}_{\text{CBSA}}}$

$$\text{=}\frac{\left[ \frac{Resident COVID19 deaths in {facility}_{i} {month}_{j}}{Occupied beds in {facility}_{i} {month}_{j}} X 100,000 \right]\text{ }}{\left[ \frac{COVID19 deaths in {CBSA}_{k} {month}_{j}}{{Population in CBSA}_{k}} X 100,000 \right]}$$
