# Supplementary material for: Interpreting COVID-19 deaths among nursing home residents in the US: The changing role of facility quality over time
Source: PLoS One. 2021 Sep 1;16(9):e0256767. doi: 10.1371/journal.pone.0256767 (PMC8409689; doi:10.1371/journal.pone.0256767)
Supplement: S1 Table — (DOCX) [file pone.0256767.s002.docx]

**S1 Table: Regression results from the two-part zero inflated negative binomial regression (full model)**^1^

| *Dependent Variable:*  NH mortality rate (per 100,000 residents | Full Model (Adjusted for all covariates)^1^ | | | | | |
| --- | --- | --- | --- | --- | --- | --- |
|  | Factors explaining mortality counts  [Count estimation] | | | Factors explaining the probability of being an excess zero outcome  [Logit estimation] | | |
|  | Coefficient  (log count) | SE | CI | Coefficient  (log odds) | SE | CI |
|  |  |  |  |  |  |  |
| *Facility-level characteristics* | | |  |  |  |  |
| Quality |  |  |  |  |  |  |
| Main effect | -0.015 | 0.018 | -0.050, 0.020 | 0.064*** | 0.021 | 0.023, 0.105 |
| Interaction Effect | 0.0008 | 0.003 | -0.005, 0.007 | -0.013*** | 0.004 | -0.020, -0.006 |
| Ownership |  |  |  |  |  |  |
| Main effect | ns | ns | ns | ns | ns | ns |
| Interaction Effect | ns | ns | ns | ns | ns | ns |
| Time (Month) | ns | ns | ns | 0.606*** | 0.094 | 0.421, 0.791 |
| Size |  |  |  |  |  |  |
| Main effect |  |  |  |  |  |  |
| Less than 50 beds (Ref) |  |  |  |  |  |  |
| 50-99 beds | -0.754*** | 0.127 | -1.003, -0.506 | -1.203*** | 0.129 | -1.458, -0.948 |
| 100-199 beds | -1.226*** | 0.119 | -1.459, -0.993 | -1.852*** | 0.131 | -2.108, -1.595 |
| 200 or more beds | -1.763*** | 0.148 | -2.053, -1.473 | -2.272*** | 0.191 | -2.647, -1.898 |
| Interaction Effect |  |  |  |  |  |  |
| Less than 50 beds (Ref) |  |  |  |  |  |  |
| 50-99 beds | 0.063*** | 0.022 | 0.019, 0.107 | 0.079*** | 0.023 | 0.033, 0.123 |
| 100-199 beds | 0.065*** | 0.021 | 0.023, 0.106 | 0.126*** | 0.023 | 0.080, 0.171 |
| 200 or more beds | 0.047** | 0.023 | 0.001, 0.094 | 0.128*** | 0.026 | 0.076, 0.179 |
| Staff Shortages (duration) |  |  |  |  |  |  |
| Main effect | 0.036*** | 0.012 | 0.011, 0.060 | -0.084*** | 0.015 | -0.113, -0.054 |
| Interaction Effect | -0.001 | 0.002 | -0.006, 0.004 | 0.003 | 0.003 | -0.002, 0.008 |
| PPE Shortages (duration) |  |  |  |  |  |  |
| Main effect | ns | ns | ns | ns | ns | ns |
| Interaction Effect | ns | ns | ns | ns | ns | ns |
| %White residents |  |  |  |  |  |  |
| Main effect | 0.003** | 0.001 | 0.0004, 0.005 | 0.007*** | 0.002 | 0.002, 0.011 |
| Interaction Effect | 0.0002 | 0.0003 | -0.0003, 0.0007 | -0.002*** | 0.0003 | -0.003, -0.001 |
| %Medicaid residents |  |  |  |  |  |  |
| Main effect | ns | ns | ns | ns | ns | ns |
| Interaction Effect | ns | ns | ns | ns | ns | ns |
| Cases among residents ^3^ | 0.016*** | 0.0005 | 0.015, 0.017 |  |  |  |
| Deaths among residents from all causes other than COVID (proxy indicator for acuity) ^3^ |  |  |  |  |  |  |
| Main effect | ns | ns | ns |  |  |  |
| Interaction Effect | ns | ns | ns |  |  |  |
| *Community-level characteristics* | | |  |  |  |  |
| CBSA death rate (per 100,000 residents) - logged |  |  |  |  |  |  |
| Main effect | 0.168*** | 0.040 | 0.088, 0.247 | -0.922*** | 0.048 | -1.017, -0.826 |
| Interaction Effect | 0.013 | 0.009 | -0.004, 0.030 | 0.062** | 0.014 | 0.035, 0.089 |
| CBSA case rate (per 100,000 residents) - logged |  |  |  |  |  |  |
| Main effect | -0.023 | 0.0513 | -0.123, 0.0778 | -0.002 | 0.060 | -0.120,0.117 |
| Interaction Effect | -0.004 | 0.0109 | -0.0250, 0.0178 | -0.108*** | 0.015 | -0.137, -0.079 |
| Urban (Metro location) |  |  |  |  |  |  |
| Main effect | 0.073 | 0.067 | -0.058, 0.204 | -0.542*** | 0.086 | -0.711, -0.373 |
| Interaction Effect | -0.013 | 0.012 | -0.037, 0.011 | 0.067*** | 0.016 | 0.035, 0.099 |
| CDC Social vulnerability index |  |  |  |  |  |  |
| Main effect | ns | ns | ns | ns | ns | ns |
| Interaction Effect | ns | ns | ns | ns | ns | ns |

Note:

^1^ The full model included all covariates we presented under the Methods section.

^2^ We did not adjust for infection among nursing home staff as that would introduce simultaneity bias.

^3^ We did not include these variables in the inflate (logit) part of the regression model as there is no reason to believe that these factors would contribute to excess zero outcomes.

^4^ Abbreviations CI: Confidence Interval | SE: Standard Error (robust) | ns: not significant

^5^ * p < 0.1, ** p < 0.05, *** p < 0.01
