# Supplementary figures and images for: Interpreting COVID-19 deaths among nursing home residents in the US: The changing role of facility quality over time
Source: PLoS One. 2021 Sep 1;16(9):e0256767. doi: 10.1371/journal.pone.0256767 (PMC8409689; doi:10.1371/journal.pone.0256767)

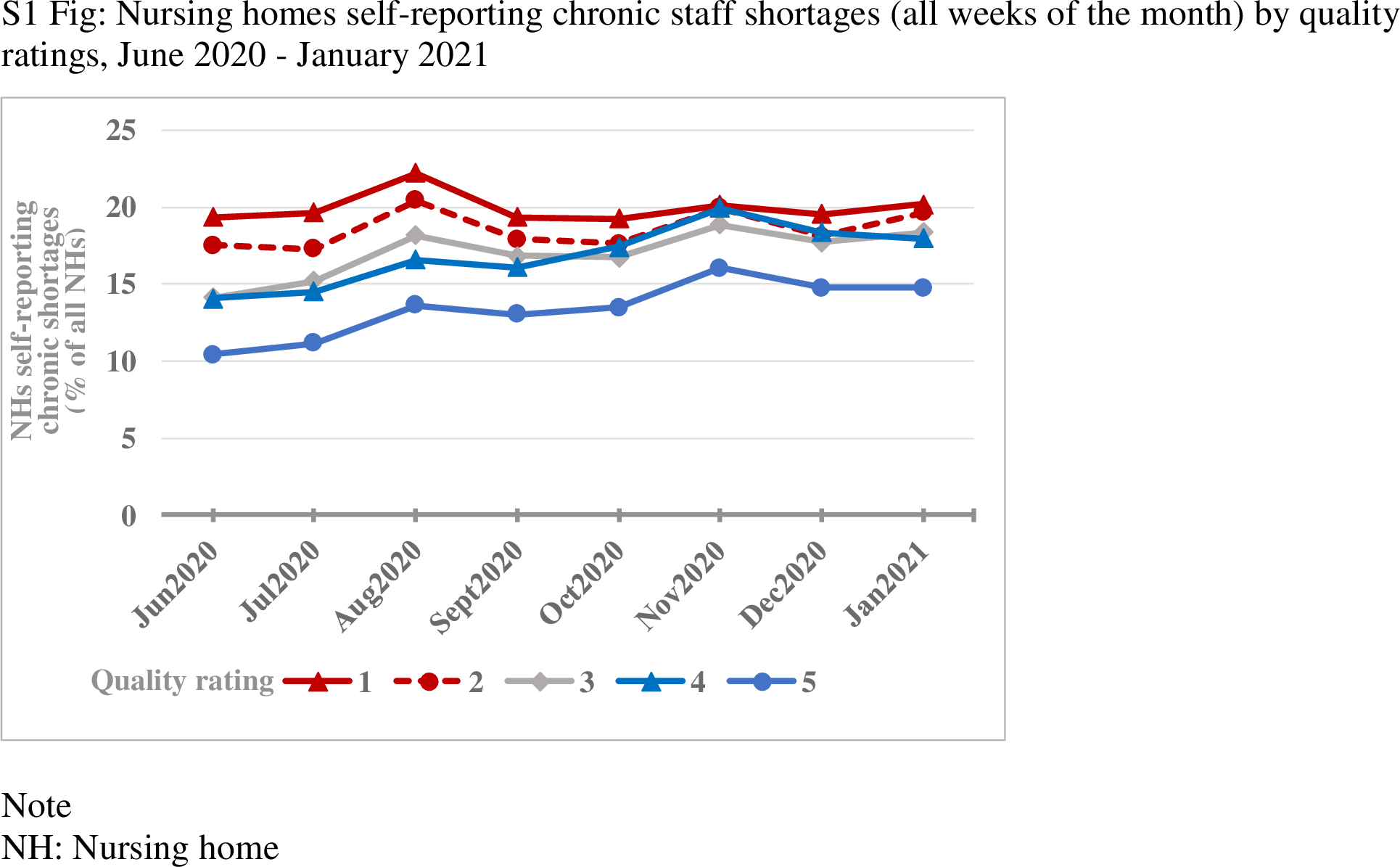

Supplement: S1 Fig — (TIF) [file pone.0256767.s003.tif]
